# Staphylococcal Food Poisoning From Cheese Products: A Narrative Review of Public Health Implications and Preventive Strategies

**DOI:** 10.7759/cureus.107857

**Published:** 2026-04-28

**Authors:** Elias Chaidoutis, Olympia Chatzimpirou, Athanasios Migdanis, Ioannis Migdanis, Antonios Papadakis, Vassiliki Pitiriga, Andreas C Lazaris, Nikolaos Kavantzas

**Affiliations:** 1 First Department of Pathology, Medical School of Athens, National and Kapodistrian University of Athens, Athens, GRC; 2 Department of Nutrition and Dietetics, University of Thessaly, Larissa, GRC; 3 Department of Gastroenterology, University of Thessaly, Larissa, GRC; 4 Division of Social Medicine, School of Medicine, University of Crete, Heraklion, GRC; 5 Department of Clinical Microbiology, Medical School of Athens, National and Kapodistrian University of Athens, Athens, GRC

**Keywords:** cheese, food safety, public health, staphylococcal enterotoxins, staphylococcal food poisoning, staphylococcus aureus

## Abstract

Staphylococcal food poisoning is a leading foodborne illness caused by the ingestion of enterotoxins produced by *Staphylococcus aureus*. A comprehensive literature search was conducted across PubMed, Scopus, and Google Scholar databases for peer-reviewed studies and official reports published between 2000 and 2026, focusing on staphylococcal enterotoxins in cheese. This review examines the epidemiology, risk factors, and microbiological aspects of *S. aureus* in cheese, emphasizing outbreaks, contamination sources, and critical points in production and storage. Inadequate milk pasteurization, starter culture failure, poor handling practices, and temperature abuse are key contributors to contamination. Surveillance data indicate a rising incidence of staphylococcal food poisoning cases linked to raw milk cheeses. Effective prevention strategies include stringent hygiene protocols across the dairy supply chain by food business operators, enhanced food safety regulations by competent authorities, and consumer training on proper cheese handling and storage to protect public health.

## Introduction and background

Cheese products have been a basic foodstuff from antiquity to the present [1]. These foods have high nutritional value and are significant for the food industry and consumer behavior [2,3]. Cheese products remain popular worldwide due to their unique organoleptic qualities and dense nutritional profile, being a major source of calcium, phosphorus, and proteins [4,5]. While these nutritional components contribute to the overall health profile of dairy products [6], from a food safety perspective, this specific biochemical matrix - characterized by high lipid and protein concentrations - serves as a protective medium that enhances the environmental persistence and thermostability of staphylococcal enterotoxins (SEs) [1]. Greece, where the production of cheese products is based on a centuries-long tradition, is among the top countries in terms of per capita cheese consumption [7].

Cheese and other foods are not sterile products [8]. The microbial flora of dairy cows may come from infected dairy animals carrying microorganisms that enter raw milk and affect production processes, and the processing environment largely determines their hygiene and safety [8-10]. Studies have demonstrated the presence of toxigenic bacterial strains in nonheat-treated dairy products, including cheese [10,11]. Notably, even pasteurized products can be infected throughout the production process because of anthropogenic contamination (e.g., contamination from personnel or contaminated equipment) [2,11]. Factors, such as storage or transport at high temperatures, increase the proliferation of microorganisms and the production of toxins even in foods that are considered safe [11].

*Staphylococcus aureus* is a key risk factor for food safety because of its ability to produce enterotoxins [12,13]. Staphylococcal food poisoning (SFP) has been recorded as one of the most widespread foodborne intoxications in the world [13,14]. Τhe clinical syndrome is caused by the ingestion of preformed thermostable SEs present in foods, rather than the ingestion of viable bacteria and their subsequent colonization of the host [15]. The preformed enterotoxins maintain biological activity even after the viable pathogen population has declined, owing to their particular adaptability and thermostability [16]. The public health burden remains substantial; while in the United States, the annual incidence is estimated at approximately 241,188 cases [12], recent data from the European Union (European Food Safety Authority (EFSA)/European Centre for Disease Prevention and Control (ECDC), 2024) confirm its persistence, reporting 207 outbreaks and 2,268 human cases for the year 2023 [17].

The purpose of the present paper is to highlight the involvement of cheese products as vectors of SFP transmission, the epidemiological data of diseases related to cheese consumption, and the preventive measures needed to protect public health.

## Review

Methodology

This narrative review is designed to provide a comprehensive synthesis of SFP in cheese products. To ensure transparency, a structured search strategy was implemented across PubMed, Scopus, and Google Scholar for the period 2000-2026. Relevant sources were identified using combinations of the keywords "*Staphylococcus aureus*", "staphylococcal food poisoning", "staphylococcal enterotoxins", "cheese", "food safety", and "public health". Inclusion criteria focused on peer-reviewed original research and reviews on enterotoxin stability in cheese, epidemiological reports from EFSA, CDC, and WHO, and studies published in English or Greek. Exclusion criteria included studies not involving cheese products, non-peer-reviewed abstracts, and articles with insufficient microbiological data. This narrative approach was chosen as it facilitates the integration of complex and heterogeneous data across different scientific fields. 

Epidemiology of SFP

Characteristics of the Microorganisms

Staphylococci are spherical gram-positive bacteria (cocci) that appear as irregular aggregates throughout microscopic examination and form grape-like clusters [18,19]. The name combines the prefix “staphylo-” from Ancient Greek and the suffix “coccus” [16,18]. While modern genomic taxonomy recognizes an increasing number of species, staphylococci are traditionally categorized for practical and diagnostic purposes by their production of the enzyme coagulase [20,21]. *S. aureus* is the primary coagulase-positive staphylococcus, with other species, such as *Staphylococcus intermedius* and *Staphylococcus hyicus*, also capable of producing enterotoxins [21]. In contrast, *Staphylococcus epidermidis*, *Staphylococcus saprophyticus*, and *Staphylococcus haemolyticus* are coagulase-negative staphylococci [20]. They are usually found in humans (skin and nasal cavity), forming part of the normal flora, but they are also present in animals, air, dust, sewerage, water, and environmental surfaces [16,18,22]. They are also resistant to infected abscess dressings, clothes, and fabrics [22,23]. In 1880, they were isolated and identified as etiological factors of various diseases, including intestinal toxicity. In approximately 1900, the toxin that produces the bacterium was determined to be the cause of SFP, and it was named enterotoxin because it targets the intestines [24].

*S. aureus* growth and the production of enterotoxins in foods require the presence of an organic source of nitrogen (e.g., amino acids) and a source of energy (carbohydrates), vitamins (thiamine, riboflavin, niacin), and metals. Additionally, environmental conditions (e.g., temperature, pH) must be favorable for its growth [25]. Bacteria grow optimally in the presence of oxygen; however, they can grow under anaerobic conditions, although at a slower pace [26]. Growth is hindered when the concentration of CO₂ in the atmosphere reaches 80% and is resistant to drought conditions [18,20,23]. The ability of *Staphylococcus* to grow under low water activity (a_w_) confers a competitive advantage in dry foods [20]. Notably, it grows even at high NaCl concentrations. Microorganisms are easily killed at cooking or pasteurization temperatures, but their resistance increases in dehydrated products with a high fat content [27]. Its ability to grow at low water activity and high salinity allows it to outcompete natural lactic acid bacteria (LAB) in salted cheese varieties, while its survival across a wide temperature range highlights the critical risk posed by even minor temperature abuses during storage and distribution. The *S. aureus *growth conditions are summarized in Table 1.

**Table 1:** Staphylococcus aureus growth conditions Sources: [16,20,25]

Growth conditions	Limiting conditions	Ideal conditions
Water activity	0.83-0.99	0.99
pH	4.2-9.3	7.0-7.5
% NaCl	≥25%	0-10%
Temperature (°C)	6-48°C	37°C

While staphylococcal risk in artisanal cheeses is often linked to the pathogen's ability to survive traditional manufacturing processes and the initial microbiological quality of raw milk [9], data from North America and Asia frequently associate outbreaks with post-pasteurization handling errors or the co-occurrence of the pathogen in processed milk supplies [12,15]. Furthermore, the risk profile shifts significantly based on the cheese variety; soft cheeses, characterized by higher moisture content and slower acidification, present a more permissive environment for enterotoxin production [11], compared to hard, long-ripened varieties where the rapid decline in pH and thermal stress serve as natural barriers [9].

The SEs produced by *S. aureus* are exotoxins, which are natural proteins with low molecular weights [18]. They are detected through specialized antibodies and include many types (SEA, SEB, SEC1, SEC2, SEC3, SED, SEE, SEG, SEH, SEI, SEJ, SEK, SEL, and SEM) that demonstrate structural similarities [16,24]. The type F enterotoxin has not been identified, as the name was assigned to a protein with no enterotoxin activity [24]. Enterotoxin A (SEA) is the predominant toxin produced most frequently [21]. The production of SEA is often associated with food poisoning outbreaks, and SED found in contaminated dairy products, in strains isolated from foodstuffs, is usually dominant either individually or in combination with other types of SE [16]. SE production has also been observed in other species of *Staphylococcus* (e.g., *S. intermedius*), but most cases of SFP are related to *S. aureus *[16,19].

The highest production of SE is observed under aerobic conditions [24]. SE thermostability, a critical property for food safety, lies in the ability of SE to withstand boiling for 30 minutes to one hour, maintaining the ability to induce disease even in sufficiently cooked foods [26,27,28]. The decoupling of pathogen viability from toxin persistence, as highlighted by the high thermostability of SEs, represents a fundamental food safety challenge. The SE production conditions are summarized in Table 2. 

**Table 2:** Staphylococcal enterotoxin production conditions by Staphylococcus aureus Sources: [27,28]

Growth conditions	Limiting conditions	Ideal conditions
Water activity	0.87-0.99	0.98
pH	4.5-9.6	7-8
Temperature (°C)	10-48°C	40-45°C
% NaCl	0%	0-10%

The growth of *S. aureus* in food depends on the presence of competitive microorganisms (e.g., LAB starter cultures) as well as environmental factors (e.g., handling and storage), which largely determine the production of toxins [25]. *S. aureus* is a poor competitor in complex microbial communities; therefore, foods with absent or disrupted natural microflora (cooked or salted) favor its growth [29].

SFP Disease Characteristics

SFP is an acute poison induced by the consumption of thermostable SE in foods contaminated with *S. aureus* [14,16,18]. It is characterized by rapid-onset gastroenteritis that appears typically within one to six hours, although the incubation period can range from 30 minutes to eight hours, depending on host sensitivity and toxin dose [28]. Symptoms primarily involve projectile vomiting, nausea, and abdominal cramps, often accompanied by diarrhea. SEs are resistant to peptic enzymes, such as pepsin and trypsin, which explains how they retain their activity after consumption, and they are highly heat-resistant [16,21,30,31]. The disease is caused by a very small amount of SE (<1 μg), although clinical studies in volunteers have shown that 20-25 μg of SEB causes symptoms [32-34]. Epidemiological data show that <200 ng SEA is sufficient in vulnerable populations [35,36]. Research results have shown that even amounts of 20-100 ng SEA are capable of causing symptoms in vulnerable adult populations [37]. Compared with healthy adults, infants and patients with underlying diseases are more at risk of SFP, with the risk of contracting the disease being determined mainly by the potential of exposure [38].

SEs are classified as medium risk for food safety, as they usually cause short-term diseases without particular complications [39,40]. The disease has a low mortality rate of <0.02%, while deaths are occasionally observed in vulnerable age groups (infants and the elderly) [28,38]. The effect of SFP varies depending on hygiene conditions, with estimations ranging from >1 to >100 cases per 100,000 people annually [28]. The peak of this seasonal disease occurs at the end of summer due to high temperatures [41]. The human body is the main carrier of microorganisms [22]. *S. aureus* colonizes the nasopharynx and skin of 35-40% of the healthy population [28,42] and may enter the chain of cheese products through contaminated milk from animals with mastitis [8,37,43].

SFP Variation in the USA and Europe

SFP remains a significant public health issue worldwide and is particularly associated with the consumption of cheese contaminated with *S. aureus*. Studies show that SFP cases are often reported in food business establishments such as restaurants, catering services, and home handling and consumption [44-46].

In the European Union (2022), SE was responsible for 137 outbreaks, 2,199 cases, 148 hospitalizations, and four deaths, with higher contamination rates in ready-to-eat meat and cheese [17]. According to the 2023 European Union One Health Zoonoses Report, published by EFSA and ECDC in 2024, *S. aureus* toxins were associated with 207 food-borne outbreaks and 2,268 cases, with Portugal and Romania reporting *S. aureus* toxins as the leading causative agents of food-borne outbreaks [47]. The true burden remains unknown for many reasons, such as the unreliable information provided by patients to health care professionals, the misdiagnosis of the disease owing to its clinical similarities to other food poisoning diseases (e.g., vomiting caused by the vomiting toxin *Bacillus cereus*), the insufficient collection of samples for laboratory analyses and the avoidance of medical attention due to the short-term or mild course of the disease [19,48,49]. Recent data highlight the importance of monitoring *S. aureus* in traditional cheeses, where the presence of enterotoxin genes has been well documented. A study of traditional Minas cheeses revealed that a significant proportion of samples contained *S. aureus*, particularly strains expressing genes encoding nonclassical enterotoxins, indicating the need for enhanced surveillance and preventive measures in dairy production chains in both the United States and Europe [50,51]. 

The observed variations in SFP incidence reflect broader shifts in food safety surveillance and consumer behavior. In the USA, the relative stability of reported outbreaks, despite high estimated case numbers [12], suggests that most cases are linked to isolated post-processing handling errors in highly standardized industrial environments. European trends are more influenced by the diversity of artisanal production, where raw milk quality and animal health remain significant variables [17,28]. These variations are often more indicative of changes in diagnostic sensitivity, such as the increased adoption of molecular enterotoxin detection, rather than actual changes in pathogen prevalence. Furthermore, the persistent seasonal peaks in late summer underscore that the environmental temperature remains a dominant factor in the SFP risk profile globally [12,28,41].

SFP Due to Cheese Products

SFP is usually associated with the consumption of dairy products, including cheese products [8]. Scientific studies have shown that *S. aureus* is frequently observed in dairy products, with significant enterotoxin production in several cheese varieties [49]. Studies focusing on cheese production have identified several factors that contribute to *S. aureus* contamination of cheese. It has been shown that traditionally prepared cheese involving direct handling can be particularly vulnerable to *S. aureus* because of the use of unpasteurized milk and inappropriate food handling practices [44,50,52]. Specific studies have shown that *S. aureus *was responsible for a high percentage of cases of SFP in France from approximately 1999-2000, emphasizing that dairy products, especially cheese, continue to contribute significantly to foodborne illnesses [51,53]. In a study of Italian raw milk cheese production, enterotoxin strains of *S. aureus* were identified that harbor the classic sea-see enterotoxin, indicating potential for food poisoning [54]. Furthermore, ineffective sample collection and inadequate diagnostic protocols complicate the accurate estimation of SFP incidence rates [51].

In the EU (1993-1998), dairy products were responsible for 4.8% of SFP cases, with variations, at 26% of cases in France, 13.9% of cases in Spain, and 3% in the United Kingdom [16]. In Germany, dairy products are associated with 3.9% of outbreaks, with cheese being responsible for 0.1%, whereas in Italy, *S. aureus* is responsible for 1.8% of all cases, with cheese products being responsible for 3.6%. In Portugal, microorganisms are responsible for 9.9% of the outbreak, with cheese products being involved in up to 1.7% of the cases. In some cases, such as an SFP outbreak linked to soft cheese in Switzerland, strong evidence of SEs, including SEA and SED, has been reported [50,52]. The production of SEA and SED can occur over the entire growth range of *S. aureus*, whereas SEB and SEC exhibit greater sensitivity to decreased a_w_ and high acidity. Industrial production systems, which typically rely on pasteurization, face primary risks from post-processing contamination often linked to equipment biofilms or human handling during large-scale packaging and distribution [12]. Conversely, traditional and artisanal cheese-making environments, particularly those utilizing raw milk, are more vulnerable to the initial microbiological quality and the animal health status (e.g., subclinical mastitis) [9,17]. Furthermore, the risk is inherently linked to the cheese variety; soft and fresh cheeses, with their higher moisture content and slower acidification, provide a more permissive medium for bacteria proliferation compared to hard, long-ripened varieties. The documented outbreaks summarized in Table 3 reflect these diverse risk factors across various geographical regions and production scales [10,30,52,55-67]. 

**Table 3:** Staphylococcal food poisoning outbreaks involving cheese products in different countries

Country	Year	Number of cases	Product	Toxin	Milk processing	Reference
USA	1884	Unspecified	Cheese	Unspecified	Unspecified	Hennekinne et al. [13]
USA	1958	200	Cheese	Unspecified	Raw	Johnson et al. [55]
USA	1965	Unspecified	Cheese	Unspecified	Unspecified	Zehren et al. [56]
Canada	1977	12	Cheese	Unspecified	Unspecified	Johnson et al. [55]
Canada	1980	62	Curd	SEA and SEC	Unspecified	Todd et al. [57]
USA	1981	16	Cheese	Unspecified	Pasteurized	Altekruse et al. [58]
England	1983	2	Cheese	Unspecified	Pasteurized	Barrett et al. [59]
France	1983	20	Cheese	SEA and SED	Raw	De Buyser et al. [60]
Scotland	1984	27	Cheese	SEA	Raw	Bone et al. [61]
England	1988	155	Cheese	Unspecified	Unpasteurized	Maguire et al. [10]
Brazil	1994	7	Cheese	SEH	Unspecified	Pereira et al. [62]
France	1997	140	Cheese	Unspecified	Raw	Kérouanton et al. [63]
France	1998	62	Cheese	Unspecified	Raw	Kérouanton et al. [63]
France	1998	37	Semi-hard cheese	Not detected	Raw	Kérouanton et al. [63]
Brazil	1999	378	Soft cheese	SEA, SEB, and SEC	Raw	Simeão Do Carmo et al. [64]
France	2001	4	Soft cheese	SEA	Unspecified	Kérouanton et al. [63]
France	2001	46	Semi-hard cheese	SED	Raw	Kérouanton et al. [63]
France	2002	104	Sheep’s milk cheese	SEA	Raw	Kérouanton et al. [63]
France	2009	23	Cheese	SEE	Unpasteurized	Ostyn et al. [65]
Switzerland	2014	14	Soft cheese	SEA and SED	Raw	Johler et al. [30]
Italy	2018	Unspecified	Creamy cheese	Unspecified	Unspecified	Börekçi et al. [66]
France	2019	Unspecified	Creamy cheese	SEA and SEB	Unpasteurized	Börekçi et al. [66]
Brazil	2021	Unspecified	Local artisanal cheese	SEA	Raw	Cardozo et al. [52]
Poland	2022	Unspecified	Fresh soft cheese	SEA	Raw	Szczuka et al. [67]

The genetic diversity of *S. aureus *strains and their resistance to traditional cheese products highlight the need for strict hygiene control [37,68]. According to EFSA (2023), 0.2-0.7% of cheese samples in Spain, Italy, and Romania, within the framework of official controls, were found to be positive for SE at the wholesale and retail level as well as at the stage of production [47].

The behavior of the pathogen in cheese products

Milk contamination by *S. aureus* can occur in infected dairy herds, food handlers, and those with incorrect hygiene practices throughout production [8,69]. Bacterial strains of human origin produce SE more frequently than those isolated from cattle do [16]. The initial microbiological quality of milk emerges as a critical factor, as cheese production from raw milk does not involve pathogen inactivation methods (e.g., heat treatment). Other risk factors include the temperature control of raw milk, acidification rate (pH decrease), curd formation, length of cheese maturation, salt concentration (NaCl), a_w_, pH value, and the use of nitrates [33].

The initial concentration of *S. aureus* in raw milk and its ability to grow during the early phase of fermentation (before the starter culture predominates) determine whether it will reach levels (>10⁵ CFU/g) capable of producing SE that remain active in the end product [2,27]. In fermented foods (e.g., cheese), *S. aureus* growth is partially limited by the type and activity of LAB (e.g., *Lactobacillus lactis*, *Lactobacillus helveticus*, *Streptococcus thermophilus*), lactic acid production (decrease in pH< 5.3), and the secretion of bacteriocins [25,70-72]. The addition of starter cultures (e.g., *Leuconostoc cremoris*) may inhibit the growth of *S. aureus* even in contaminated samples, according to experiments in various types of cheese [73]. The factors that affect SE production, *S. aureus *growth, and SE production in cheese products include the amount of LAB present at high concentrations (>10⁷ CFU/g), pH values < 5.0, which inhibits the production of toxins, and NaCl concentrations >10%, which helps to inhibit the growth of microorganisms, and heat treatment [74]. Once SE is produced, it remains active for months. On the other hand, staphylococci are eliminated within a time ranging from one week to two months, depending on their initial amount and the type of cheese [8].

In fresh cheeses (Cottage, Baker's, Cream, Mizithra, Ricotta), the high amount of LAB (>10⁸ CFU/g) inhibits *S. aureus* growth even at initial contamination levels of 10³-10⁵ CFU/g, increasing the risk of fermentation failure or the low initial activity of LAB [16]. In soft cheeses (Camembert, Brie, Feta, etc.), unsuccessful fermentation with *S. aureus* >10⁴ CFU/g may lead to SE production and therefore production issues [8,16]. In semihard/hard cheeses (Edam, Cheddar, Kefalotyri, etc.), unsuccessful fermentation and a slow decrease in pH may lead to the potential growth of *S. aureus* in the case of initial contamination >10³ CFU/g, as the pH value does not inhibit bacterial growth [71]. Cheeses with surface mold growth are more vulnerable if the initial contamination exceeds 10³ CFU/g; in interior mold-ripened cheeses (e.g., Roquefort, Gorgonzola, Stilton, Danablue, and blue cheese), experimental studies have not shown SE production, possibly because interior mold-ripened cheeses do not provide a favorable environment for *S. aureus *growth. This is probably due to the combined inhibitory activity of *Penicillium* spp. and starter bacteria [16, 25]. The heat treatment of some processed cheese products (cheese sauce, spread, etc.) at temperatures ranging from 80-85°C is mainly responsible for their safety, as it inactivates pathogenic microorganisms [16,74].

Preventive measures regarding the production of cheese products

*S. aureus *control requires the implementation of strict hygiene measures at all stages of production (from primary production to retail). Hazard analysis critical control point (HACCP) systems focus on the identification and control of critical parameters (e.g., temperature, pH, and microbe content) to inhibit SE production.

At the primary production stage, one of the most significant factors for SE production is the initial *S. aureus* concentration in milk (>10³ CFU/mL) [27]. Food business operators (FBOs) must take measures to prevent the contamination of water supplies and stocks of feedingstuffs, pests (insects, birds, etc.), soil contamination, dust particles, and contaminated water droplets. FBOs should also pay particular attention to fecal contamination and adhere strongly to personal hygiene rules [33,75]. Animal health and veterinary control are also important for preventing potential mastitis, as contaminated cow milk contains relatively high concentrations of *S. aureus *[16]. Temperature control is also important by cooling milk to a temperature of <4°C within four hours after milking and avoiding temperature variations when the product is transported to the production unit [69].

Inappropriate environmental conditions may lead to contamination or induce *S. aureus* growth and SE production [27,76]. Food processing areas, including piping, floor drains, dishwashing stations, conveyor systems, air handling units, packaging, and cold storage areas [75,77,78], have been identified as potential sources of contamination. All the required good hygiene practices (GHPs), such as proper waste management and pest control to prevent cross-contamination, must be adhered to [76,79].

Milk pasteurization should be carried out at 72°C for 15 seconds (closed system) or at 63°C for 30 minutes (open system) according to European legislation [79,80]. Pasteurization significantly reduces bacterial counts, and adding a LAB culture may inhibit residual pathogens by creating an acidic environment [8,75,79]. The risk of fermentation failure as a result of insufficient lactic acid production (pH>5.3) remains significant for the production of *S. aureus *[16,75]. Appropriate ripening conditions are necessary to promote biochemical changes (e.g., sugar fermentation and amino acid production) [18,81]. The presence of antibiotics or bacteriophages, as well as the presence of detergent residues, may disrupt LAB starter cultures [18,27,69].

pH is a means of controlling pathogen growth on a product [8,16]. The pΗ of the main Greek cheeses according to the Food and Beverage Code ranges from 4.1 for soft cheeses with a creamy texture, with intermediate values of 4.3 for soft brine cheeses, 5.1 to 5.7 for semihard and hard cheeses, and up to 5.9 for soft whey cheeses [79,82]. Deviations from the optimum pH (5.2-5.3) in fresh cheese may indicate fermentation failure, which makes effective monitoring of *S. aureus* [69,83] necessary. The addition of sodium chloride reduces the a_w_ value to inhibit microbial growth, although *S. aureus* can still produce toxins at salt concentrations of up to 10% (a_w_ = 0.92) [16,81,84].

The food safety management system must involve control measures to prevent high levels of *S. aureus* growth (>10⁴ CFU/g) and, most importantly, the maintenance of appropriate storage temperatures [33]. After production, cheese products are maintained at cooling temperatures (≤4°C) until they are placed on the market [79]. The Greek Food and Beverage Code specifies their maintenance at temperatures up to 2°C [82]. The end product coating after ripening helps protect cheese from microorganisms, normal wear during transport and distribution, and contamination at retail [81].

In the primary phase of cheese production, FBOs must take into account that a large amount of *S. aureus* may grow in milk before curdling, and when the level of cheese acidity is not satisfactory within the first 24 hours, resulting in the production of SEs. Notably, if enterotoxin is already produced, it remains active in the product even after microorganisms have been eliminated [27]. In the next stages of the production process, the number of microorganisms usually decreases, making their simple count in the end product inadequate to indicate potential SE presence [16]. Therefore, the European Union has established criteria for semihard and hard cheeses and soft whey cheeses, as they provide favorable conditions for *S. aureus* growth because of their high a_w_ and increased pH [16]. The limits regarding coagulase-positive staphylococci and SE are presented in Table 4. 

**Table 4:** Legislative limits according to Regulation (European Commission) 2073/2005 on microbiological criteria in food

Type of cheese	Staphylococcus aureus (CFU/g)	SE (25 g)
Cheese made from raw milk	≤10⁵	Not detectable
Soft cheeses (pasteurized)	<10²	Not detectable
Semi-hard/hard cheeses	<10³	Not detectable

FBOs need to comply with microbiological criteria in food established by European Legislation and with validation and verification requirements for HACCP-based procedures through microbiological tests [85,86]. ISO 6888-1:2021 is a horizontal method for the enumeration of coagulase-positive staphylococci (*S. aureus* and other species) [87]. Τhe diagnostic landscape has significantly expanded with the integration of molecular techniques. PCR-based methods allow for the rapid identification of *S. aureus* and the comprehensive screening of the enterotoxin genes [50]. While immunoassays are essential for confirming the presence of the active toxin in the food matrix, molecular tools provide critical insights into the toxigenic potential of the isolated strains [37]. This genomic approach is indispensable for modern monitoring strategies and outbreak investigations, as it allows for the detection of non-classical SE genes that may not be covered by standard immunoassay kits [50,51].

Compared with strains isolated from cattle, human microbial strains have been identified as more frequent enterotoxin producers [16]. Food handling by asymptomatic workers or insufficient implementation of GHPs throughout production may lead to outbreaks of outbreaks [78]. Effective staff training is a determining factor for the successful implementation of a HACCP plan. Training must include clear instructions for monitoring critical control points (CCPs), and management should ensure adequate time, materials, and equipment for proper training. Education and experience in FBOs play key roles in the enforcement of good manufacturing processes, ensuring safe cheese-making, and maintaining high hygiene standards [75,76]. Consumers must be adequately informed to understand the information regarding proper food handling and follow the instructions that accompany the products to prevent the occurrence of food-borne diseases [78].

Limitations

While this narrative approach facilitates the integration of complex and heterogeneous data, we acknowledge potential selection bias as a limitation compared to systematic review protocols. However, the synthesis provided here offers a critical overview providing actionable insights for FBOs and regulatory authorities.

## Conclusions

SFP is recognized as a major contributor to the global burden of foodborne diseases, frequently ranking among the leading causes of bacterial outbreaks reported by surveillance systems. Despite the extremely low mortality index (<0.02%), SFP continues to pose a major public health issue, particularly in vulnerable population groups, such as infants and elderly people. As disease incidence persists, implementing GHPs throughout the whole production chain of cheese products and the proper use of LAB starter cultures is crucial. Actionable recommendations for FBOs include implementing GHPs throughout the production chain, with emphasis on temperature control, milk pasteurization, and HACCP principles.

For competent authorities, compliance with European Legislation and the execution of official controls remain key preventive measures. Furthermore, to bridge existing monitoring gaps, the implementation of new technologies for the rapid detection of toxin strains, such as PCR and immunosensors, should be prioritized. These integrated measures, combining traditional hygiene with modern molecular tools, are essential for the effective prevention of SFP outbreaks associated with cheese products.
